# Ultrasensitive Fluorescence Sensing of Chlorpyrifos Using Core–Shell Au@Ag Nanoparticle-Enhanced Inner Filter Effect on g-C_3_N_4_

**DOI:** 10.3390/bios16070376

**Published:** 2026-07-09

**Authors:** Mengli Wang, Yuanyuan Xia, Yulei Li, Lifen Chen, Kunyan Wang, Shuangshuang Wu, Yuelan Zhang

**Affiliations:** Jiaxing Key Laboratory of Molecular Recognition and Sensing, College of Biological, Chemical Sciences and Engineering, Jiaxing University, Jiaxing 314001, China

**Keywords:** electrochemiluminescence, Au@Ag, biosensing, Chlorpyrifos

## Abstract

In this work, we developed a novel, ultrasensitive fluorescence sensing platform for determination of organophosphorus pesticides (OPs), using chlorpyrifos as a representative model analyte. The sensing strategy was constructed upon the key inner filter effect (IFE) between graphitic carbon nitride (g-C_3_N_4_) nanosheets and silver-coated gold core–shell nanoparticles (Au@Ag NPs). Initially, gold nanoparticles (Au NPs), silver nanoparticles (Ag NPs), and Au@Ag NPs were successfully synthesized, and their fluorescence quenching efficiencies toward g-C_3_N_4_ were systematically evaluated. Owing to the superior spectral overlap with the fluorescence emission of g-C_3_N_4_, Au@Ag NPs exhibited the most obvious quenching effect and were thereby selected as the optimal quencher for sensor fabrication. Then, acetylcholinesterase (AChE) catalyzed the hydrolysis of acetylthiocholine (ATCH) into thiocholine. The generated thiocholine then induced aggregation of Au@Ag NPs via electrostatic and Ag-S interactions, which reduced the IFE efficiency and ultimately restored the fluorescence of g-C_3_N_4_. In contrast, the presence of chlorpyrifos effectively inhibits AChE activity, thereby suppressing ATCH hydrolysis and the subsequent aggregation of Au@Ag NPs. The fluorescence intensity of g-C_3_N_4_ was quenched by Au@Ag NPs and the signal was low. Under optimal experimental conditions, the response signal was found to be proportional to chlorpyrifos (CPF). This work presents a rapid, cost-effective, and highly sensitive approach for CPF residue analysis, holding great potential for applications in food safety monitoring and environmental surveillance.

## 1. Introduction

Chlorpyrifos (CPF), a representative broad-spectrum organophosphorus pesticide (OPs), demonstrates remarkable efficacy against a diverse range of agricultural pests and has been widely used in global agricultural production [1]. However, growing concerns regarding its potential health hazards have attracted considerable scientific attention [2]. The International Agency for Research on Cancer (IARC) has classified chlorpyrifos as a Group 2A carcinogen [3]. Its primary mechanism involves the inhibition of acetylcholinesterase (AChE) activity in the nervous system, thereby disrupting normal neurotransmission and inducing acute poisoning symptoms in exposed individuals [4,5]. Furthermore, prolonged or repeated exposure may lead to chronic damage to multiple organ systems, including the nervous and reproductive systems [6]. Given the widespread application, extensive environmental residues, and potential health risks of OPs, the development of rapid, highly sensitive, and reliable analytical methods for the trace-level detection of chlorpyrifos has become increasingly urgent.

To date, the commonly used analytical methods for CPF include high-performance liquid chromatography (HPLC) [7], capillary electrophoresis (CE) [8], gas chromatography (GC) [9], chromatography-mass spectrometry (GC-MS/LC-MS) [10], and enzyme-linked immunosorbent assay (ELISA). However, these conventional methods typically involve labor-intensive pretreatment procedures, high operational costs, and prolonged analysis time, which limited their practical applicability. In the past few years, fluorescence-based sensing strategies have emerged as a highly promising platform in biosensing because of their inherent advantages, including fast response, high sensitivity, excellent reproducibility, and operational simplicity [11]. The inner filter effect (IFE) is an optical phenomenon in which coexisting light-absorbing substances absorb the excitation or emission light of fluorophores, thereby attenuating their intrinsic fluorescence [12]. Owing to the distinct advantages of label-free detection, high sensitivity and simple and flexible operation, IFE has shown great potential for applications in the field of environmental monitoring [13].

g-C_3_N_4_ is a metal-free p-type semiconductor polymer with a characteristic aromatic C–N framework [14]. Benefiting from its excellent resistance to acidic, alkaline, and organic solvents, g-C_3_N_4_ possesses outstanding thermodynamic and chemical stability [15]. As an emerging and promising fluorescent nanomaterial, g-C_3_N_4_ effectively overcomes the inherent shortcomings of conventional fluorescent probes, such as high cost, poor stability, and environmental toxicity, thus exhibiting tremendous application potential in fluorescence sensing analysis [16]. Moreover, noble metal nanoparticles exhibit unique localized surface plasmon resonance (LSPR) characteristics, with remarkable molar extinction coefficients and tunable broadband absorption properties, which enable them to serve as superior light-absorbing quenchers [17,18]. The significant spectral matching between g-C_3_N_4_ and noble metal nanoparticles endows the composite system with efficient IFE quenching behavior, allowing for the precise modulation of g-C_3_N_4_ fluorescence signals [19]. The efficient inner filter effect (IFE) of Au NPs on fluorescent probes has been demonstrated as a successful strategy in spectrofluorometry [20,21]. Using g-C_3_N_4_ as a fluorescent probe and gold nanoparticles (Au NPs) as an absorber probe, a simple, environmentally friendly, and sensitive dual-signaling (fluorometric and colorimetric) method for detecting organophosphorus pesticides (OPs) was initially developed [22]. Furthermore, in another way, a high-sensitivity and high selectivity analytical approach for OPs quantification using silver nanoparticles modified with graphitic carbon nitride was developed, which provides a reliable technical alternative for the efficient and trace-level determination of Ops [23]. To the best of our knowledge, the fluorescent sensor for OPs based on the g-C_3_N_4_ and Au@Ag has never been reported.

This study developed a highly sensitive and specific fluorometric sensing platform for the quantitative detection of organophosphorus pesticides (OPs). g-C_3_N_4_ was utilized as a fluorescent donor with stable intrinsic fluorescence, while Au@Ag acted as efficient fluorescence absorbers and quenchers. Acetylthiocholine (ATCh) can undergo catalytic hydrolysis by acetylcholinesterase (AChE) to produce thiocholine (TCh) [24]. The sulfhydryl groups (-SH) in TCh possess a strong and specific binding affinity for silver atoms on the surface of Ag@Au nanoparticles, facilitating the formation of Ag–S covalent bonds and electrostatic adsorption. This specific chemical interaction leads to the aggregation of dispersed Ag@Au nanoparticles, which reduced the IFE quenching efficiency and ultimately allows the effective recovery of fluorescence. Nevertheless, the presence of chlorpyrifos can significantly inhibit the catalytic activity of AChE [25]. The inactivation of AChE suppresses the hydrolysis of ATCh and blocks the generation of TCh, which prevents the aggregation of Au@Ag nanoparticles. The original IFE quenching effect is therefore retained, leading to a concentration-dependent decrease in the fluorescence intensity of the sensing system. On this basis of this fluorescence response, the constructed platform can realize the sensitive and quantitative detection of organophosphorus pesticides.

## 2. Materials and Methods

### 2.1. Materials and Instruments

The chlorpyrifos standard stock solution (10 μg/L in methanol) was diluted serially to prepare working solutions of the required concentrations. Acetylthiocholine (ATCh, 20 mM) was freshly prepared in double-distilled water and used within 3 h to ensure stability. Both CPF and acetylthiocholine (ATCh) were purchased from Shanghai Aladdin Biochemical Technology Co., Ltd. (Shanghai, China) Acetylcholinesterase (AChE, 1 unit/mL) obtained from Shanghai Macklin Biochemical Co., Ltd. (Shanghai, China), was dissolved in Tris-HCl buffer (pH 7.5, 0.02 mM) prior to use. Melamine, anhydrous ethanol, silver nitrate (AgNO_3_), sodium borohydride (NaBH_4_, 96%), and trisodium citrate (C_6_H_5_O_7_Na_3_·2H_2_O) were all purchased from Sinopharm Chemical Reagent Co., Ltd. (Shanghai, China). All chemicals used in this study were of analytical reagent grade. All solutions were prepared using double-distilled deionized water. Before use, all glassware was thoroughly cleaned by successive rinsing with the use of aqua regia and double-distilled water (three times each) to eliminate potential contamination. To characterize the morphologies of the nanoparticles, transmission electron microscopy (Talos F200X, Thermo Fisher Scientific, Waltham, MA, USA) was employed. An Ultraviolet-visible spectrophotometer (UV-2700, Shimadzu, Shanghai, China) was used to record the ultraviolet-visible absorption spectrum, A fluorescence spectrophotometer (Shanghai Lengguang Technology, F97pro, Shanghai, China) was used to obtain the fluorescence spectra.

### 2.2. Preparation of g-C_3_N_4_

g-C_3_N_4_ was synthesized via a direct thermal treatment according to previously reported methods [26]. Specifically, 10 g of melamine was placed in a covered alumina crucible and heated to 600 °C at a rate of 3 °C/min and maintained at this temperature for 2 h. After cooling to room temperature, the resulting powder was obtained. The obtained product was then subjected to ultrasonic treatment in water prior to use.

### 2.3. Preparation of Ag NPs

A 100 mmol/L AgNO_3_ solution was placed in an ice-water bath at 5 °C under vigorous stirring. Subsequently, 250 µL of 100 mmol/L trisodium citrate and 6 mL of 5 mmol/L sodium borohydride (NaBH_4_) solution were added. The reaction proceeded rapidly, and the mixture turned from colorless to light purple. The mixture was stirred continuously for another 30 min, and the product was stored in the dark for 24 h prior to use.

### 2.4. Preparation of Au NPs

10 mL of a 33 mM sodium citrate solution was introduced into the flask. The mixture was maintained at a temperature of 137 °C while being continuously stirred for 50 min. Subsequently, 1 mL of a 25 mM chloroauric acid solution was rapidly injected into the vortex center of the stirring mixture. After one minute, 5 mL of a 0.1 M tris(hydroxymethyl)aminomethane (Tris) solution was added in one portion. Then, 1 mL of the 25 mM chloroauric acid solution was added into the vortex center, and the reaction continued at 120 °C for another 30 min. Upon completion of this step, an additional 1 mL of 25 mM chloroauric acid solution was added under the same conditions. After the reaction was completed, the resulting product was transferred to a clean container and stored at room temperature in a dark place.

### 2.5. Synthesis of Au@Ag Composite Nanoparticles

A 5 mL solution of gold nanospheres was mixed with 10 µL of 4-ATP and then centrifuged at 6000 rpm for 10 min. After removing the supernatant, the precipitate was resuspended in 10 mL of ultrapure water. The resulting dispersion was heated to boiling in an oil bath at 120 °C under vigorous stirring, and then 1.25 mL of 1% sodium citrate solution was rapidly added. After 3 min, a total volume of 250 µL of AgNO_3_ solution was added quickly (25 µL every 30 s for a total of 10 additions) and the mixture was heated with stirring for an additional 15 min. The reaction was then terminated, and the mixture was allowed to cool to room temperature with continuous stirring.

### 2.6. Fluorescence Detection of Chlorpyrifos

To investigate the fluorescence quenching effect of Au@Ag NPs and to validate the inner filter effect (IFE) between g-C_3_N_4_ and Au@Ag NPs, the fluorescence intensities of mixed solutions containing a fixed amount of g-C_3_N_4_ and varying concentrations of Au@Ag NPs were measured. Specifically, 400 µL of g-C_3_N_4_ solution (3.0 mg/mL) was mixed with different volumes of Au@Ag NPs solution and the mixtures were diluted to a final volume of 600 µL using PBS buffer (10 mM, pH 8.0). The final concentrations of Au@Ag NPs were set at 0, 0.3, 0.6, 1.0, 2.0, 3.0, 4.0 and 5.0 nM. Fluorescence emission spectra of the systems were recorded at 440 nm under an excitation wavelength of 365 nm. For detection of CPF, acetylcholinesterase (AChE, 6 µL, 1.0 U/mL) was incubated with various concentrations of chlorpyrifos in PBS buffer (10 mM, pH 8.0) at 37 °C for 15 min. Subsequently, 400 µL of g-C_3_N_4_ solution (3.0 mg/mL) and 100 µL of Au@Ag NPs solution (3.0 nM) were added. Finally, each mixture was supplemented with 9 µL of acetylthiocholine chloride (ATCh, 1.0 mM). The resulting solution was diluted to a final volume of 600 µL with PBS buffer and thoroughly mixed. Fluorescence emission spectra and UV-Vis absorption spectra were measured separately.

## 3. Results

### 3.1. Characterization of Ag NPs, Au NPs and Au@Ag NPs

**Figure 1:**
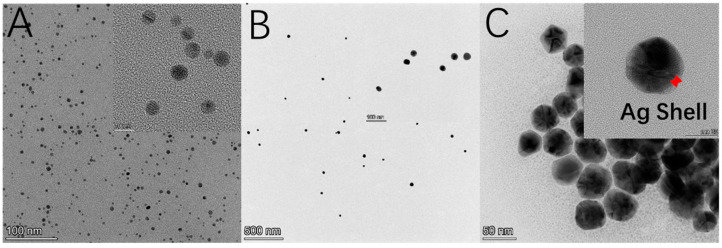
TEM images of (**A**) Ag NPs, (**B**) Au NPs and (**C**) Au@Ag NPs. The Ag Shell is labeled with a red arrow.

TEM analysis revealed that Ag NPs were well-dispersed with a uniform spherical shape and an average diameter of ~6 nm (Figure 1A), verifying the successful preparation of high-quality Ag nanospheres with stable geometry. In comparison, Au NPs (Figure 1B) exhibited a larger average particle size of 24 nm while maintaining a monodisperse spherical morphology. For Ag@Au core–shell nanoparticles (Figure 1C), the transparent outer region is the silver shell—an Ag shell (3–5 nm) was uniformly deposited on its surface—while the dark inner part is the gold core, confirming the successful fabrication of the desired core–shell architecture.

### 3.2. Spectral Characterization of Ag NPs, Au NPs and Au@Ag NPs, and C_3_N_4_

**Figure 2:**
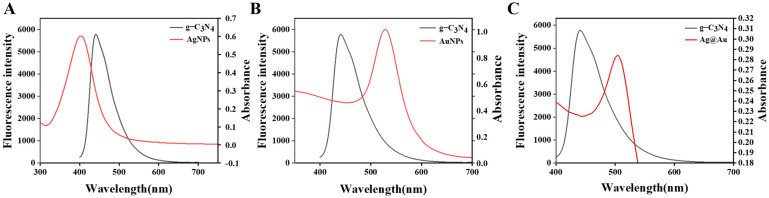
(**A**–**C**) The absorption spectra of Ag NPs, Au NPs and Au@Ag NPs, and the fluorescence emission spectrum of g-C_3_N_4_.

To verify the feasibility of the inner filter effect (IFE) between g-C_3_N_4_ and various noble metal nanomaterials (Ag NPs, Au NPs, and Ag@Au core–shell NPs), we systematically analyzed their UV–vis absorption and fluorescence emission spectra (Figure 2). The particle sizes of Ag NPs and Au NPs adopted in this sensing system were selected according to the widely used conditions reported in the previous literature [22]. Among the three materials, Ag NPs and Au NPs both exhibited partial spectral overlap with the g-C_3_N_4_ emission, resulting in a relatively weak IFE quenching effect. In contrast, the Au@Ag core–shell NPs demonstrated stronger broadband absorption and optimal spectral overlap with the g-C_3_N_4_ fluorescence, attributed to the synergistic optical properties of the Ag core and Au shell. Consequently, this architecture enables highly efficient IFE-based quenching of g-C_3_N_4_ fluorescence, ensuring high sensitivity and stable performance for the proposed fluorescence sensing platform in organophosphorus pesticide detection.

### 3.3. The Influence of Concentration of Ag NPs, Au NPs and Au@Ag NPs on Fluorescence Intensity of g-C_3_N_4_

**Figure 3:**
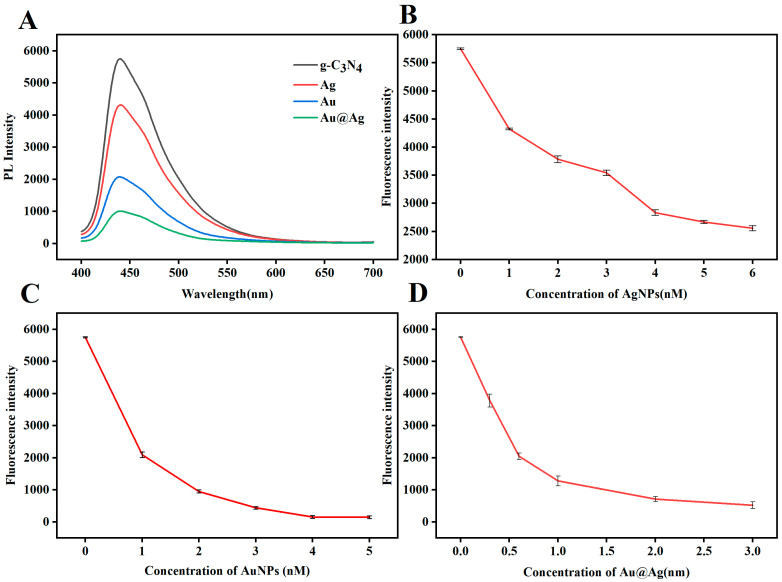
(**A**) Fluorescence between g-C_3_N_4_ and Au@Ag. (**B**–**D**) Influence of Ag NPs, Au NPs and Au@Ag concentration on fluorescence intensity of g-C_3_N_4_; three independent parallel experiments were performed.

The fluorescence quenching efficiency of g-C_3_N_4_ by Au NPs, Ag NPs, and Au@Ag NPs was systematically investigated. Figure 3A compares the fluorescence emission spectra of g-C_3_N_4_ after interaction with Ag NPs, Au NPs, and Au@Ag NPs. All three nanoparticles effectively quenched the fluorescence of g-C_3_N_4_, with the quenching efficiency following the order Au@Ag NPs > Au NPs > Ag NPs. To further quantify the quenching behavior, the effect of the concentration of nanoparticles on fluorescence intensity was evaluated (Figure 3B–D). At a concentration of 1 nM, Au@Ag NPs exhibited significantly higher quenching efficiency toward g-C_3_N_4_ than Ag NPs and Au NPs, demonstrating optimal quenching performance. This enhanced quenching is mainly attributed to the Au@Ag core–shell architecture, which combines the plasmonic properties of both Au and Ag, resulting in greater spectral overlap between its absorption band and the emission band of g-C_3_N_4_. This increased overlap strengthens the inner filter effect and facilitates interfacial charge transfer, leading to more efficient fluorescence quenching. Consequently, Au@Ag NPs were selected as the optimal functional material for constructing a high-performance g-C_3_N_4_-based fluorescence sensing platform for organophosphorus pesticide detection.

### 3.4. Feasibility of the Proposed Biosensor

**Figure 4:**
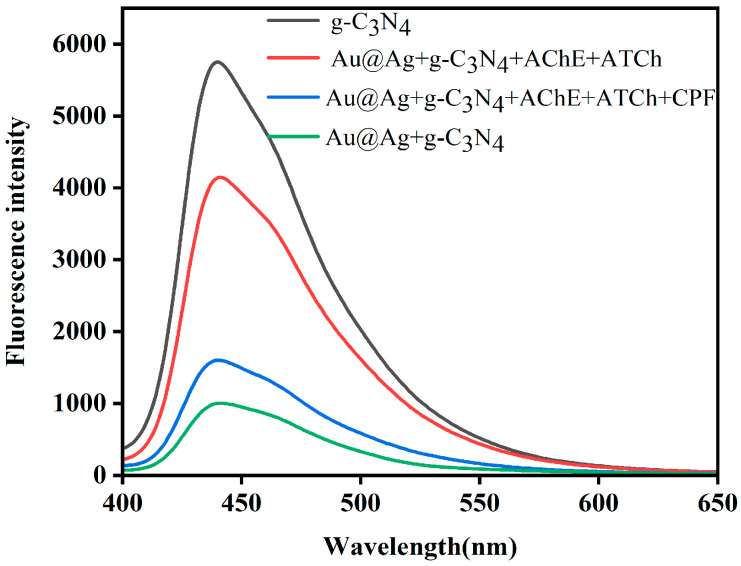
The fluorescence emission spectrum of g-C_3_N_4_ interacting with different substrates.

g-C_3_N_4_ exhibits excellent fluorescence photostability and water dispersibility, as well as strong fluorescence emission, rendering it an ideal fluorescent substrate for constructing fluorescence sensing platforms. Figure 4 presents the fluorescence emission spectra of g-C_3_N_4_ under various conditions. Upon the introduction of Au@Ag nanoparticles, the fluorescence intensity decreased dramatically due to the inner filter effect. When acetylcholinesterase (AChE) and acetylthiocholine (ATCh) are sequentially added to the g-C_3_N_4_/Au@Ag system in the absence of organophosphorus pesticides (OPs), a significant fluorescence recovery is observed. This recovery is attributed to the enzymatic hydrolysis of ATCh, which is catalyzed by AChE to produce large amounts of thiocholine (TCh). The sulfhydryl groups (–SH) of TCh readily form stable Ag–S covalent bonds with the metal sites on the Au@Ag surface, inducing the aggregation of Au@Ag nanoparticles. This aggregation increases the distance between the nanoparticles and g-C_3_N_4_, weakens their interfacial interaction, and consequently eliminates the IFE quenching effect, restoring the fluorescence. However, in the presence of chlorpyrifos (as a model OP), the fluorescence intensity remains at a relatively low level without notable recovery. This is because chlorpyrifos specifically binds to the active centers of AChE, occupying the catalytic sites and irreversibly inhibiting its enzymatic activity. The inactivated AChE fails to catalyze the hydrolysis of ATCh to generate TCh, thus preventing the aggregation of Au@Ag nanoparticles. As a result, the nanoparticles remain well dispersed in the system, and the IFE-based quenching of g-C_3_N_4_ fluorescence persists. Therefore, by monitoring the suppression of fluorescence recovery, the proposed platform enables highly sensitive detection of organophosphorus pesticides.

### 3.5. Optimization of Experimental Conditions

**Figure 5:**
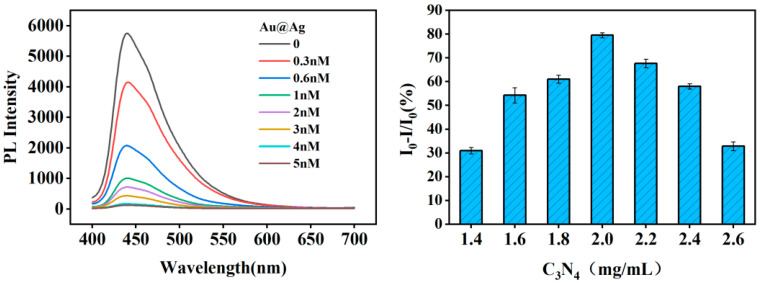
The fluorescence intensity of different concentrations of g-C_3_N_4_ in the presence of Au@Ag NPs. And the fluorescence intensity of g-C_3_N_4_/Au@Ag NPs under different concentrations of g-C_3_N_4_.

To achieve the optimal and stable sensing performance for the fluorescence detection of chlorpyrifos, a series of critical experimental parameters that significantly affect the fluorescence quenching efficiency caused by the inner filter effect between g-C_3_N_4_ and Au@Ag NPs were systematically optimized. As depicted in Figure 5, it can be observed that the fluorescence intensity of g-C_3_N_4_ decreased gradually as the concentrations of Au@Ag ranged from 0 to 5.0 nM; maximum quenching was observed when the concentration of Au@Ag reached 1.0 nM. Thus, the fluorescence of g-C_3_N_4_ could be controlled by the Au@Ag via IFE process and the optimal concentration of Au@Ag was confirmed to be 1nM. On this basis, the influence of g-C_3_N_4_ concentration on the sensing performance was further investigated in detail. The fluorescence reduction efficiency varied significantly with the change in g-C_3_N_4_ concentration. With the gradual increase in g-C_3_N_4_ concentration, the fluorescence quenching efficiency increased continuously and reached the maximum value at the g-C_3_N_4_ concentration of 2.0 mg/mL. When the g-C_3_N_4_ concentration exceeds 2.0 mg/mL, excessive g-C_3_N_4_ nanosheets will cause material aggregation in the solution, thereby leading to a decrease in fluorescence quenching efficiency. Consequently, 2.0 mg/mL was selected as the optimal working concentration of g-C_3_N_4_.

### 3.6. Performance of Proposed Biosensor

**Figure 6:**
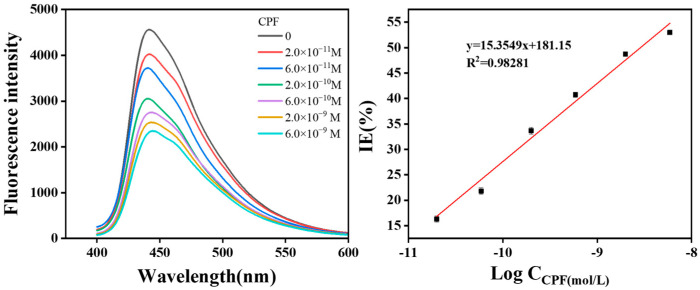
Fluorescent curves of the biosensor towards different concentrations of targets: 6.0 × 10^−9^, 2.0 × 10^−9^, 6.0 × 10^−10^, 2 × 10^−10^, 6.0 × 10^−11^, 2.0 × 10^−11^ mol/L, And the Calibration curve of the sensor response to chlorpyrifos concentrations. The parallel experiments were carried out three times.

Under the optimal experimental conditions, the developed sensing platform was employed for quantitative detection of chlorpyrifos. As shown in Figure 6, the fluorescence intensity of the g-C_3_N_4_/Au@Ag nanocomposite exhibited a concentration-dependent quenching response upon the addition of chlorpyrifos over the concentration range of 2.0 × 10^−11^ to 6.0 × 10^−9^ M. The correlation between the fluorescence quenching efficiency and the logarithm of chlorpyrifos concentration was described by the regression equation y = 15.3549Log(C) + 181.1 with a correlation coefficient (R^2^) of 0.9828, indicating excellent linearity and reliability for quantitative analysis. The substantial quenching effect demonstrates the high sensitivity of the proposed sensing system, which can be attributed to the efficient energy transfer mediated by the inner filter effect between g-C_3_N_4_ and Au@Ag nanoparticles upon target recognition. The limit of detection (LOD) calculated by the formula LOD = 3σ/k was 1.73 × 10^−13^ M (6.1 × 10^−8^ mg/kg) and the practical measurement detection of limit is 2 × 10^−11^ M. which is significantly lower than the national standards specified maximum residue limit of methyl parathion in food (0.02 mg/kg). The limit of detection of this work was compared with previous reports, demonstrating a superior and lower LOD (Table S1) [27,28,29,30]. The results demonstrate that this method possesses excellent high sensitivity and is suitable for trace analysis.

### 3.7. Selectivity and Stability of the Proposed Biosensor

To evaluate the selectivity of the sensor, three organophosphorus pesticides (OPs), namely paraoxon, malathion and fenitrothion, were tested. As shown in Figure S3, high quenching efficiencies were observed upon the addition of these three OPs. This phenomenon originates from the P=O group, which can effectively suppress the activity of acetylcholinesterase (AChE). In contrast, non-OP interferents including nitrobenzene, phenol and p-methoxyphenol barely altered the sensor signals and get small quenching efficiencies. These observations verify the outstanding anti-interference ability of the sensing platform. Furthermore, the reproducibility of the sensor was investigated. Six parallel measurements were performed under identical experimental conditions. The ECL signal remained steady with a relative standard deviation (RSD) of 3.8%, demonstrating favorable stability of the constructed sensor.

### 3.8. Application of ECL Bacterial Sensor in Real Samples

The standard addition method was employed to evaluate the suitability of the biosensor for detecting chlorpyrifos in actual samples. Briefly, 10 g of lettuce was thoroughly washed, and the lettuce was homogenized using a juicer to prepare vegetable extracts. The resulting extracts was blended with 100 mL of 10 mM PBS buffer (pH 8.0). After 10 min of ultrasonication, the mixture was centrifuged at 8000 rpm for 10 min, and the supernatant was collected for subsequent measurements. Various concentrations of chlorpyrifos were added to lettuce leaf extracts to evaluate the precision and accuracy of the sensing method in practical samples. As listed in Table 1, the spiked recoveries were 99.6%, 98.8% and 104%, with relative standard deviations (RSD) of 0.32%, 2.53% and 4.32%, respectively. All these results demonstrate that the as-constructed sensing system exhibits excellent application potential for the determination of chlorpyrifos residues in real samples.

## 4. Conclusions

In this work, a comparative investigation was conducted to evaluate the fluorescence quenching performance of various synthesized nanospheres toward two-dimensional g-C_3_N_4_ nanosheets. Experimental results confirmed that Au@Ag core–shell nanospheres exhibited the most remarkable fluorescence quenching ability. Leveraging the inner filter effect (IFE) between Au@Ag nanospheres and g-C_3_N_4_ nanosheets, a highly sensitive fluorescence sensing platform was further constructed for the quantitative determination of chlorpyrifos. The developed biosensor exhibited excellent detection performance, with a good linear relationship established between the fluorescence intensity and the analyte concentration, yielding the regression equation: y = 15.3549 log(C) + 181.1. This platform demonstrates high sensitivity and reliability. Overall, the Au@Ag/g-C_3_N_4_ fluorescence sensor based on the IFE holds great promise for the detection of organophosphorus pesticides. This study not only provides an effective strategy for the trace analysis of chlorpyrifos and other organophosphorus pesticides in complex practical samples, but also offers a valuable design rationale for the construction of fluorescence sensors based on core–shell metal.

## Figures and Tables

**Table 1:** Recovery results of spiked chlorpyrifos in real samples.

No.	Added (ng/mL)	Mean Measured (ng/mL)	Mean Recovery (%)	RSD (%)
1	0.05	0.0498	99.6	0.32
2	0.5	0.494	98.8	2.53
3	1	1.04	104	4.32

## Data Availability

The original contributions presented in this study are included in the article/Supplementary Materials. Further inquiries can be directed to the corresponding authors.

## Supplementary Materials

The following supporting information can be downloaded at: https://www.mdpi.com/article/10.3390/bios16070376/s1, Figure S1: Particle size distribution diagram of the nanoparticles; Figure S2: The TEM and mapping of g-C_3_N_4_; Figure S3: The selectivity of the sensor. Table S1: Compare this work with other previous work.
