# Supplementary material for: Ultrasensitive Fluorescence Sensing of Chlorpyrifos Using Core–Shell Au@Ag Nanoparticle-Enhanced Inner Filter Effect on g-C3N4
Source: Biosensors (Basel). 2026 Jul 9;16(7):376. doi: 10.3390/bios16070376 (PMC13406696; doi:10.3390/bios16070376)
Supplement: Supplementary file 1 [file biosensors-16-00376-s001.zip › biosensors-4380466-supplementary.pdf]

Ultrasensitive Fluorescence Sensing of Chlorpyrifos Using Core-Shell Au@Ag Nanoparticle-Enhanced Inner Filter Effect on g-C_3N_4

Mengli Wang, Yuanyuan Xia , Yulei Li, Lifen Chen *, Kunyan Wang, Shuangshuang Wu, Yuelan Zhang*

Jiaxing Key Laboratory of Molecular Recognition and Sensing, College of Biological, Chemical Sciences and Engineering, Jiaxing University, Jiaxing, Zhejiang 314001, China.

* Correspondence: E-mail: chenlf@zjxu.edu.cn (L. Chen); E-mail: ylzhang@zjxu.edu.cn (Y. Zhang)

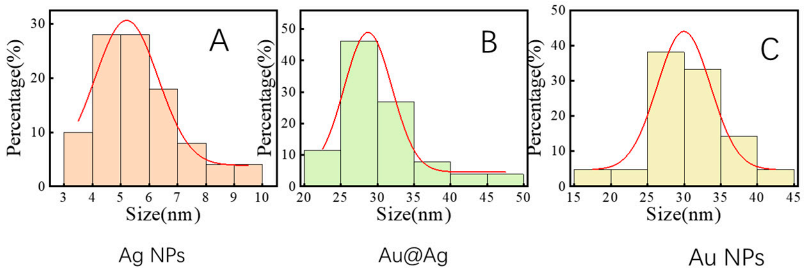

Figure S1 Particle size distribution diagram of the nanoparticles

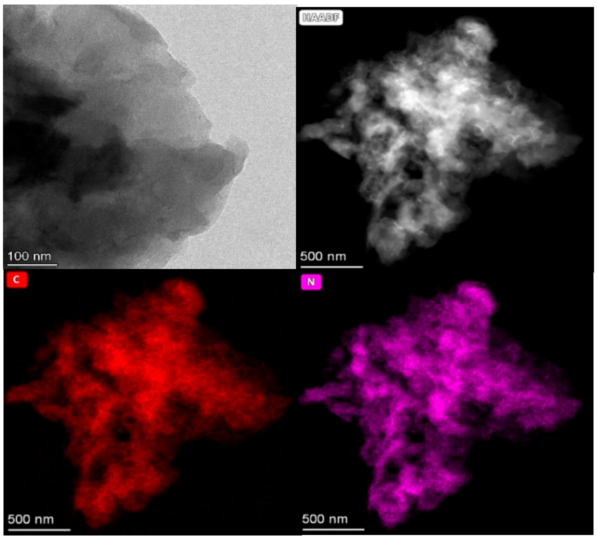

Figure S2. The TEM and mapping of g-C_3N_4

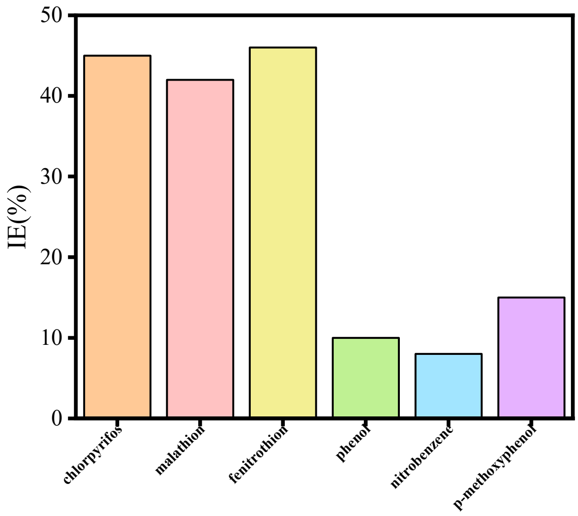

Figure S3 The selectivity of the senosr

Table S1 Compare this work with other previous work

Method	Linear Range	Detection Limit	Ref.
SERS	0.1-316 ng/mL	0.066 ng/mL	[1]
Fluorescence	2.5-250 ng/mL	1.712 ng/mL	[2]
Fluorescence	0.025-25 μg/mL	0.0008 μg/mL	[3]
Electrochemiluminescence	0.01-1000 ng/mL	0.002 ng/mL	[4]
fluorescence	6.0×10^{-9}-2.0×10-11 mol/L	(6.0×10^{-5} ng/mL) 1.73×10^{-13}	This work

References

[1] Wang, H.; Chen, Z.; Zhu, C.; Du, H.; Mao, J.; Qin, H.; She, Y.; Yan, M. An interference-free SERS-based aptasensor for chlorpyrifos detection. *Anal. Chim. Acta* **2023**, *1268*, 341398 , 10.1016/j.aca.2023.341398.

[2] Zhao, X.; Lu, Y.; Li, B.; Kong, M.; Sun, Y.; Li, H.; Liu, X.; Lu,. G. Self-ratiometric fluorescent platform based on upconversion nanoparticles for on-site detection of chlorpyrifos [J]. *Food Chem.*, **2024**, 439: 138100. DOI: 10.1016/j.foodchem.2023.138100.

[3] Zhang, L.; Chen, J.; Zhang, L.; Yu, R. Rapid detection of chlorpyrifos in miscellaneous beans based on nitrogen and phosphorus doped carbon quantum dots fluorescence probe [J]. *J. Food Compos. Anal.*, **2025**, 137: 106884. DOI: 10.1016/j.jfca.2024.106884

[4] Song, L.; Zhang, Q.; Min, L.; Guo, X.; Gao, W.; Cui, L.; Zhang, C. Electrochemiluminescence enhanced by isolating ACQphores in imine-linked covalent organic framework for organophosphorus pesticide assay [J]. *Talanta*, **2024**, 266: 124964. DOI: 10.1016/j.talanta.2023.124964.
